# Correlation of Plasma EGF with Striatal Dopamine Transporter Availability in Healthy Subjects

**DOI:** 10.1038/s41598-017-13771-9

**Published:** 2017-10-16

**Authors:** Kyoungjune Pak, Seunghyeon Shin, So Jung Kim, Keunyoung Kim, Bum Soo Kim, Seong Jang Kim, In Joo Kim

**Affiliations:** 1 Department of Nuclear Medicine and Biomedical Research Institute, Pusan National University Hospital, Busan, Republic of Korea; 2 Department of Nuclear Medicine and Research Institute for Convergence of Biomedical Science and Technology, Pusan National University Yangsan Hospital, Yangsan, Republic of Korea

## Abstract

We aimed to evaluate the association between plasma epidermal growth factor (EGF) and the availability of dopamine transporter (DAT) measured from ^123^I-FP-CIT single-photon emission computed tomography in healthy controls in this study. Volume of interest template was applied to measure specific binding ratios (SBRs) of caudate nucleus, putamen, and striatum representing DAT availability as follows; SBR = (target– cerebellum)/cerebellum. Plasma EGF was negatively correlated with the availabilities of both caudate nucleus (r = −0.261, p = 0.019), and putamen (r = −0.341, p = 0.002). After dividing subjects according to Apo E genotyping, DAT availability of caudate nucleus of Apo e4 non-carriers (n = 60) showed the positive correlation with cerebrospinal fluid (CSF) α-synuclein (r = 0.264, p = 0.042). Plasma EGF was negatively correlated with DAT availabilities of Apo e4 non-carriers. Further studies are needed to clarify underlying mechanisms of this phenomenon.

## Introduction

Epidermal growth factor (EGF), 6 kDa protein made up of 53 amino acids, is found at high concentrations in bile, urine, milk, and prostate fluid, at medium concentrations in tears, follicular fluid, sperm, and seminal plasma, and at low concentrations in plasma, serum, and saliva^1^. EGF is known to involve in the development of the nervous system, stimulating proliferation, migration, differentiation of neuronal cells, enhancing survival, and inhibiting apoptosis^2^. Supplement of EGF to Parkinson disease (PD) model rat prevented the dopaminergic neurodegeneration^3^. Decreased level of EGF was found in striatum of patients with PD as compared with controls in postmortem study^3^. Several studies reported that low plasma EGF was correlated with cognitive decline in PD patients and the high conversion rate to Alzheimer’s disease (AD)^4,5^.

PD is a clinical syndrome showing bradykinesia, tremor, rigidity, and postural instability. It is characterized by the loss of dopaminergic neuron of the substantia nigra, and the presence of intraneuronal cytoplasmic inclusion^6,7^. The loss of dopaminergic neuron is parallel to the level of expression of the dopamine transporter (DAT) mRNA^6^. DAT is on the presynaptic dopaminergic nerve terminal and controls dopamine levels by active reuptake of dopamine from the synaptic cleft^8^. As ^123^I-FP-CIT reflects the striatal DAT density^8^, the availability of DAT measured from ^123^I-FP-CIT single-photon emission computed tomography (SPECT) can be used in evaluating the neurodegenerative disease^9^.

Although the effect of EGF in neurodegenerative disease is well documented in previous reports, the correlation of EGF with DAT in healthy controls has not been investigated yet. Therefore, we evaluated the association between plasma EGF and the availability of DAT measured from ^123^I-FP-CIT SPECT in healthy controls in this study.

## Materials and Methods

### Subjects

Data used in the preparation of this article were obtained from PPMI database (www.ppmi-info.org/data). For up-to-date information on the study, visit www.ppmi-info.org ^10^. The study population consisted of 192 healthy controls with screening ^123^I-FP-CIT SPECT. According to PPMI criteria of healthy subjects, males or females with their age of 30 years or older at screening was included, and subjects with a neurological disorder, a first degree relative with idiopathic PD, Montreal Cognitive Assessment score of 26 or less, medications that might interfere with DAT SPECT scans, anticoagulants that might preclude safe completion of the lumbar puncture, or investigational drugs, and a condition that precludes the safe performance of routine lumbar puncture were excluded. Subjects without CSF biomarkers, plasma EGF, and Apo E genotyping were excluded. Medical history, cerebrospinal fluid (CSF), plasma EGF, and ^123^I-FP-CIT SPECT scans were downloaded. The PPMI study was approved by the local Institutional Review Boards of all participating sites (Institute for Neurodegenerative Disorders, University of Pennsylvania, University of California, Los Angeles, Coriell Institute for Medical Research, Clinical Trials Coordination Center, Laboratory of Neurogenetics; National Institute on Aging NIH, Institute for Neurodegenerative Disorders, Clinical Trials Statistical and Data Management Center, University of Iowa) and written informed consent was obtained from each subject at the time of enrollment for imaging data and clinical questionnaires. All methods were performed in accordance with the relevant guidelines and regulations.

### Cerebrospinal fluid biomarkers and Plasma EGF

CSF biomarkers of Aβ_1–42_, tau protein phosphorylated at the threonine 181 position (p-Tau_181_), and total tau were analyzed with multiplex xMAP Luminex platform (Luminex Corp, Austin, TX, USA), and Innogenetics immunoassay kits (Innogenetics/Fujirebio, Ghent, Belgium). α-synuclein were analyzed using an enzyme-linked immunosorbent assay (Covance Research Products Inc., Denver, PA, USA). Plasma levels of EGF were measured by enzyme-linked immunosorbent assay (ELISA, R&D Systems, Minneapolis, MN, USA) according to manufacturer instructions. Samples were run in duplicate and data used for this study met quality control measures for technical performance.

### Apo E Genotyping

Apo E genotyping was performed on DNA samples. Two non-synonymous single nucleotide polymorphisms, rs429358, and rs7412 were genotyped in each sample to distinguish between Apo e2, e3, and e4 alleles using TaqMan assays (Applied Biosystems, Foster City, CA, USA).

### ^123^I-FP–CIT SPECT

#### Protocol

^123^I-FP-CIT SPECT was performed during the screening visit for all subjects. SPECT scans were acquired 4 ± 0.5 hrs after injection of 111–185 MBq of ^123^I-FP-CIT. Subjects were pretreated with iodine solution or perchlorate prior to injection to block thyroid uptake. Raw data were acquired into a 128 × 128 matrix stepping each 3 or 4 degrees for the total projections. Raw projection data were reconstructed using iterative ordered subset expectation maximization with HERMES (Hermes Medical Solutions, Stockholm, Sweden). The reconstructed images were transferred to pmod (PMOD Technologies LLC, Zürich, Switzerland) for subsequent processing including attenuation correction.

#### Image analysis

Downloaded scans were loaded using pmod v3.6 (PMOD Technologies LLC, Zürich, Switzerland) with ^123^I-FP-CIT template^11^. Specific binding of ^123^I-FP-CIT regarding DAT was calculated using a region of interest analysis. A standard set of volume of interest (VOI) defining caudate nucleus, putamen, and striatum (caudate nucleus + putamen) based on the Automated Anatomical Labeling (AAL) atlas^12^. The cerebellum was chosen as a reference region. VOI template was applied to measure specific binding ratios (SBRs) of caudate nucleus, putamen, and striatum representing DAT availability as follows; SBR = (target– cerebellum)/cerebellum.

### Statistical Analysis

Normality was examined using D’Agostino-Pearson omnibus test. Spearman correlation was used to measure the relationship of SBRs with CSF biomarkers, and plasma EGF. Mann-Whitney test was applied to compare SBRs, CSF biomarkers, and plasma EGF between Apo e4 non-carriers and carriers. Statistical analyses were performed using GraphPad Prism 7 for Mac OS X (GraphPad Software Inc, San Diego, CA, USA).

## Results

### Subjects’ characteristics

81 healthy subjects (49 male, 32 female) were included in this study. Mean age was 62.3 years. Mean BMI was 26.5 kg/m^2^. Twenty-one subjects were Apo e4 carriers (25.9%). DAT availabilities in caudate nucleus (r = −0.313, p = 0.004) showed a reduction with aging as expected. When subjects were divided according to Apo e4 genotyping, Aβ_1–42_ was higher in Apo e4 non-carriers than Apo e4 carriers. However, age, sex, BMI, α-synuclein, p-Tau_181_, total tau, and plasma EGF showed no significant differences between Apo e4 non-carriers and Apo e4 carriers. Subjects’ characteristics are summarized in Table 1.

**Table 1:** Subjects’ characteristics according to Apo e4 genotyping.

Variables	Apo e4 non-carrier (n = 60)	Apo e4 carrier (n = 21)	p
Age (years)	61.1 (44.4~82.2)	65.4 (44.0~76.9)	0.1779
Sex (male/female)	37/23	12/9	0.7168
BMI (kg/m^2^)	26.1 (18.5~37.2)	25.4 (20.4~31.7)	0.7465
Cerebrospinal fluid biomarkers			
Aβ_1–42_ (pg/ml)	402.3 (213.7~641.4)	289.7 (108.6~447.3)	< 0.0001
α-synuclein (pg/ml)	1992.3 (592.6~5237.7)	2028.1 (864.1~3426.0)	0.5826
p-Tau_181_ (pg/ml)	13.9 (6.1~50.0)	13.3 (6.6~73.3)	0.5715
Total tau (pg/ml)	46.3 (21.5~129.4)	44.9 (18.4~104.7)	0.8293
Plasma EGF (pg/ml)	41.8 (0~175.1)	49.9 (0~350.0)	0.1369

^*^SD, standard deviation; BMI, body mass index; EGF, epidermal growth factor.

### Correlation of DAT availability with CSF biomarkers, and Plasma EGF

**Figure 1:**
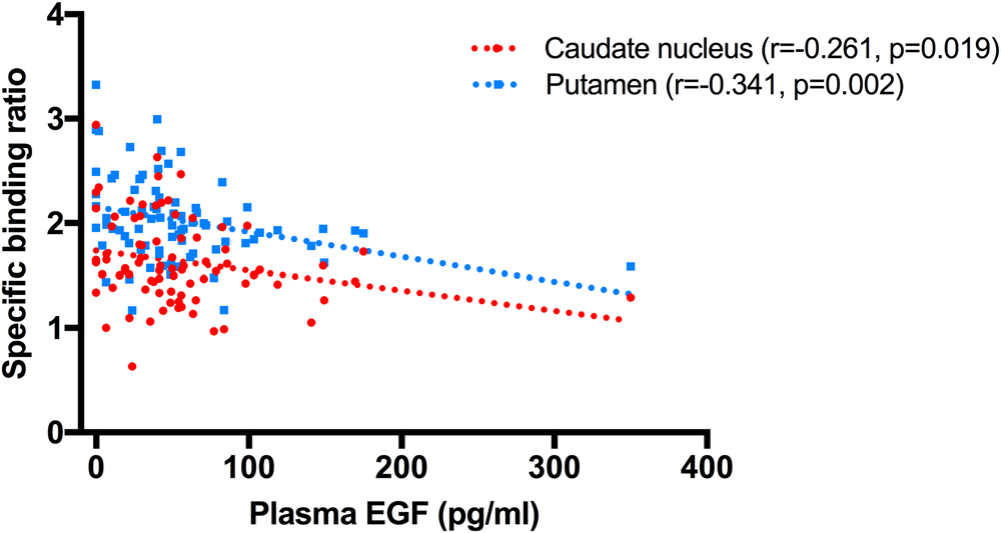
Correlation between DAT availability and plasma EGF.

**Figure 2:**
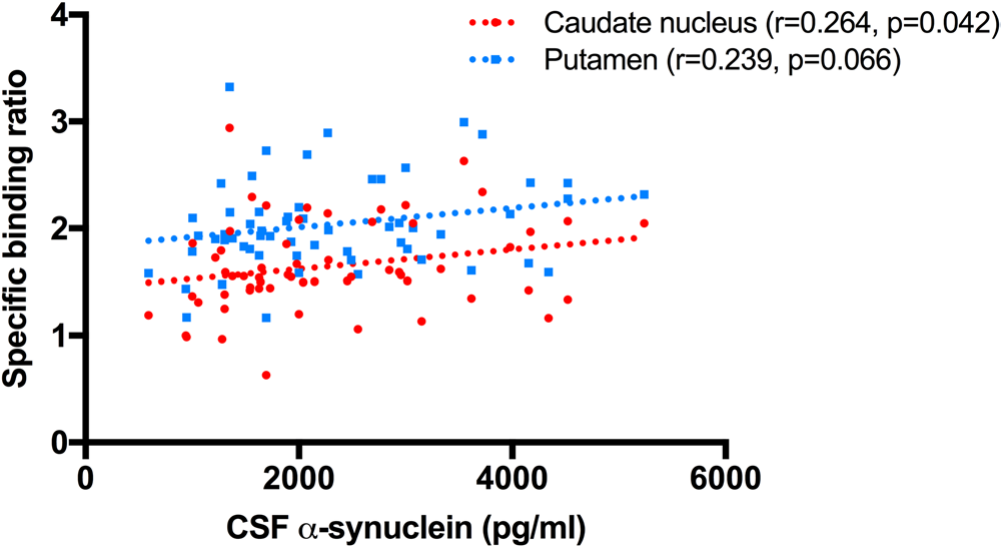
Correlation between DAT availability and CSF α-synuclein in subjects with Apo e4 non-carriers.

None of CSF biomarkers showed significant association with DAT availability (Table 2). However, plasma EGF was negatively correlated with the availabilities of both caudate nucleus (r = −0.261, p = 0.019), and putamen (r = −0.341, p = 0.002) (Fig. 1). After dividing subjects according to Apo E genotyping, DAT availability of caudate nucleus of Apo e4 non-carriers (n = 60) showed the positive correlation with α-synuclein (r = 0.264, p = 0.042), and that of putamen showed the trend with α-synuclein (r = 0.239, p = 0.066) (Fig. 2) (Table 3). Plasma EGF was negatively correlated with DAT availabilities of putamen (r = −0.368, p = 0.004) and striatum (r = −0.328, p = 0.011) of Apo e4 non-carriers.

**Table 2:** Correlations of the availability of DAT with age, BMI, cerebrospinal fluid biomarkers, and plasma EGF.

	Caudate nucleus	Putamen	Striatum
Age (years)	−0.313^*^	−0.112	−0.202^#^
BMI (kg/m^2^)	−0.049	−0.018	−0.039
Cerebrospinal fluid biomarkers			
Aβ_1–42_ (pg/ml)	0.188^#^	0.127	0.170
α-synuclein (pg/ml)	0.186^#^	0.137	0.180
p-Tau_181_ (pg/ml)	−0.118	−0.041	−0.049
Total tau (pg/ml)	0.062	0.057	0.079
Plasma EGF (pg/ml)	−0.261^*^	−0.341^*^	−0.314^*^

^*^DAT, dopamine transporter; BMI, body mass index; EGF, epidermal growth factor.

^*^P < 0.05^*^, P < 0.1^#^.

**Table 3:** Correlations of the availability of DAT with age, BMI, cerebrospinal fluid biomarkers, and plasma EGF according to Apo E genotyping.

	Apo e4 non-carrier (n = 60)			Apo e4 carrier (n = 21)		
	Caudate nucleus	Putamen	Striatum	Caudate nucleus	Putamen	Striatum
Age (years)	−0.255^*^	−0.076	−0.156	−0.275	−0.173	−0.261
BMI (kg/m^2^)	−0.038	0.042	0.006	−0.136	−0.177	−0.186
Cerebrospinal fluid biomarkers						
Aβ_1–42_ (pg/ml)	0.149	0.170	0.184	0.201	−0.008	0.127
α-synuclein (pg/ml)	0.264^*^	0.239^#^	0.276^*^	−0.031	−0.199	−0.075
p-Tau_181_ (pg/ml)	−0.069	0.028	0.025	−0.254	−0.205	−0.196
Total tau (pg/ml)	0.178	0.190	0.214	−0.250	−0.345	−0.282
Plasma EGF (pg/ml)	−0.244^#^	−0.368^*^	−0.328^*^	−0.308	−0.316	−0.387^#^

*DAT, dopamine transporter; BMI, body mass index; EGF, epidermal growth factor.

*p < 0.05*, p < 0.1#.

### Data availability

Data used in the preparation of this article were obtained from PPMI database (www.ppmi-info.org/data).

## Discussion

In this study, plasma EGF was negatively correlated with striatal DAT availability. When subjects were categorized according to Apo E genotyping, negative correlation was observed in Apo e4 non-carriers. α-synuclein was positively correlated with DAT availability in Apo e4 non-carriers.

EGF is known to have protective effect in dopaminergic neuron^13^ and stimulate the uptake of dopamine^14^. Chen-Plotkin *et al*. reported that when the PD patients were divided by quartile according to plasma EGF values, the lowest quartile of the PD patients showed the highest conversion rate to Parkinson disease dementia and they demonstrated that plasma EGF was an independent variable predicting cognitive decline in PD patients^4^. Lim *et al*. also reported that low baseline plasma EGF predicted cognitive decline in PD patients and conversion from amnestic mild cognitive impairment (MCI) to AD^5^. Jiang *et al*. demonstrated that plasma EGF was decreased in the early stage of PD and there was no significant difference of plasma EGF between advanced PD and normal control^15^. In study regarding EGF and dopamine uptake in cultured rat astrocytes, they suggested the existence of Na + -dependent and Na + -independent dopamine uptake in cultured rat astrocytes and they concluded that EGF might stimulate the expression and translocation of the extraneuronal DAT^16^. In study assessing EGF-ErbB1 action on developing midbrain dopaminergic neuron, authors showed that EGF elevated DAT level in mesencephalic cultures and they reported that EGF receptor inhibitor reduced DAT level in the striatum, nucleus accumbens, and globus pallidum in neonatal rats^17^. EGF levels in both striatum and serum of patients with chronic schizophrenia were reduced comparing with those of normal controls in postmortem study^18^. Laakso *et al*. showed that striatal DAT availability measured by^18^F-CFT was reduced in patients with chronic schizophrenia as compared with normal controls^19^. Thus, we expected that the DAT availability would be decreased if the level of plasma EGF was decreased. Unlike our expectations, negative correlation between plasma EGF and DAT availability was observed in healthy subjects. There was no relevant study to explain this negative correlation.

α-synuclein, 14 kDa protein, consists of three domains with N-terminal lipid-binding α-helix, amyloid-binding central domain, and C-terminal acidic tail^20,21^, which has a function in suppression of apoptosis, regulation of glucose levels, modulation of calmodulin activity, chaperone activity, and regulation of dopamine biosynthesis^20^. Also, it has been known to be associated with neurodegenerative disease such as PD, dementia with Lewy bodies, MSA, and AD^21^. Especially in PD, α-synuclein interacts with tubulin, parkin, dopamine receptor, synphilin-1, phospholipase, and small ubiquitin related modifiers^20^. Previous studies showed that α-synuclein was not different between Apo e4 carriers and Apo e4 non-carriers in normal subjects, MCI, and AD, consistent with this study^22,23^. However, molecular linkage between Apo E and α-synuclein was demonstrated in one study using A30P and A53T transgenic mice. α-synuclein induces neuronal degeneration leading to Apo E deposition in spinal cords, astrocytes, and activated microglia of transgenic mice^24^. Astrocyte-secreted Apo E reduced α-synuclein uptake, and the effect was seen in Apo e4, followed by e3, and e2^25^. Wersinger *et al*. reported that α-synuclein reduced DAT activity by recruitment of DAT from plasma membrane to cytoplasm in their *in vitro* study^26^. The decreased activity of DAT was caused by reduced dopamine uptake velocity, not by decreased DAT expression^27^. Kovacs *et al*. showed that the density of DAT, identified by immunochemistry, inversely correlated with the density of α-synuclein in the substantia nigra of patients with Lewy body disease and PD^28^. However, Bellucci *et al*. demonstrated that transgenic mice producing human α-synuclein had increased levels of striatal DAT^29^.

Apo E has three isoforms which are known to Apo e2, Apo e3, and Apo e4^30^. Among them, Apo e4 is known to be the greatest risk factor for AD, followed by Apo e3, contrary to protective effect of Apo e2^31^. Apo E has a major role in metabolism of Aβ, which is abundant in brain of Apo e4 carriers than Apo e4 non-carriers^31^. In this study, CSF Aβ_1–42_ was higher in Apo e4 non-carriers than Apo e4 carriers. Consistent with this study, Prince *et al*. showed decreased amount of CSF Aβ in Apo e4 carriers in both AD and normal control groups^32^. α-synuclein attenuated the effect of EGF by showing decreased luciferase activity in a study by Iwata *et al*.^33^. Therefore, Apo e4 non-carriers may have higher CSF Aβ_1–42_, CSF α-synuclein, and CSF α-synuclein leading to both lower plasma EGF and higher DAT availability.

This is the first study that investigated the association between Apo E genotyping, Aβ_1–42_, α-synuclein, plasma EGF, and DAT availability in healthy controls. However there are several limitations in this study. First, subjects included in this study was collected from PPMI database. Second, the number of subjects was small in Apo e4 carriers. Third, as PPMI database was collected from multiple institutes, the difference in image acquisition may affect the results.

In conclusion, plasma EGF was negatively correlated with DAT availabilities of Apo e4 non-carriers. DAT availability was positively correlated with α-synuclein in Apo e4 non-carriers. Further studies are needed to clarify underlying mechanisms of this phenomenon.
